# Fluorination Effects in Hexagonal XY (X, Y = C, Ge, Si, Sn; X ≠ Y) Monolayers and vdW Heterostructures: Insights from *Ab Initio* Calculations

**DOI:** 10.1021/acsomega.6c04011

**Published:** 2026-09-01

**Authors:** Renato B. Pontes, Danilo P. Kuritza, Rafael Barbosa, José E. Padilha

**Affiliations:** † Instituto de Física, 67824 Universidade Federal de Goiás, Campus Samambaia, Goiânia, Goiás 74690-900, Brazil; ‡ Departamento de Física, 42487 Universidade Estadual de Maringá, Maringá, Paraná 87020-900, Brazil; § Campus Avançado Jandaia do Sul, Universidade Federal do Paraná, Jandaia do Sul, Paraná 86900-000, Brazil

## Abstract

By combining hybrid *ab initio* electronic-structure calculations that include spin–orbit coupling with phonon-spectrum calculations and molecular-dynamics simulations, we systematically investigate a family of hexagonal XY and fluorinated XYF_2_ monolayers (X, Y = C, Si, Ge, Sn; X ≠ Y). All compounds are confirmed to be dynamically and thermally stable, with fluorination being exothermic and further enhancing lattice robustness. Fluorination universally drives a transition from planar sp^2^ to buckled sp^3^ structures, which strongly affects the electronic structure. For instance, CSi, CGe, and CSn already display sizable gaps (3.28, 2.85, and 1.96 eV, respectively) that shift slightly upon fluorination to 3.21, 2.97, and 2.27 eV. In contrast, narrow-gap systems such as SiGe (0.19 eV) and SiSn (0.41 eV) exhibit pronounced bandgap openings to 1.43 and 1.02 eV, respectively, corresponding to relative increases of ∼653 and ∼149%. GeSn, however, remains a narrow-gap semiconductor (0.33 → 0.28 eV), showing little sensitivity to functionalization. We further investigate the corresponding van der Waals bilayers and trilayers and determine their absolute band-edge positions relative to the vacuum level to evaluate their potential for photocatalytic water splitting. For CSi+CSiF_2_, SiSn+SiSnF_2_, and GeSn+SiSnF_2_ heterobilayers, we identify both type-II and type-III alignments. In CSi-based trilayers, fluorination drives a systematic transformation from a graphitic sp^2^ lattice to a fully diamondized sp^3^ lattice, driving substantial hybridization changes and a pronounced reshaping of the band structure.

## Graphical abstract

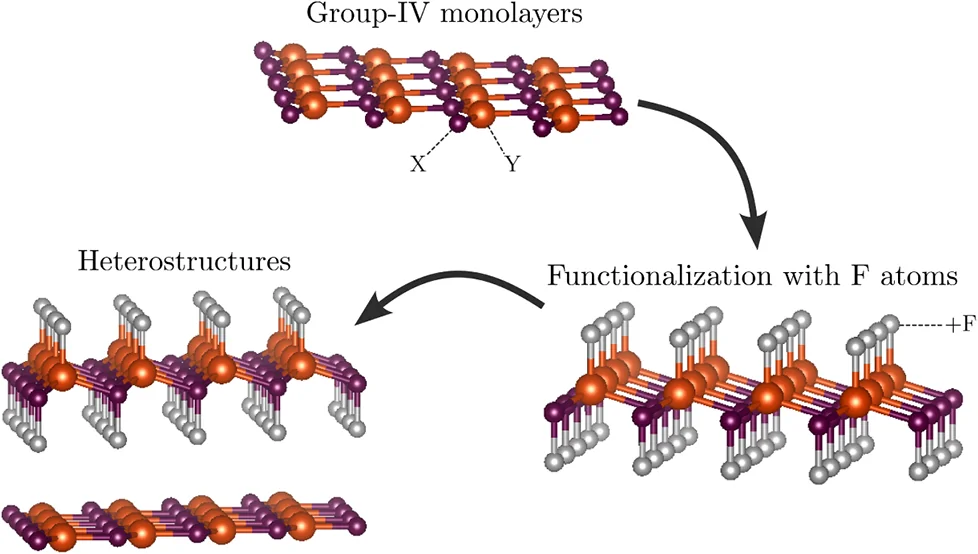

## Introduction

Nanoscience has revolutionized the field of materials research by providing a wide platform to design, explore and manipulate matter at the atomic and molecular scale.^1,2^ Among the diverse range of nanomaterials, graphene, a two-dimensional carbon allotrope, has emerged as a prominent candidate due to its exceptional mechanical, electrical, optical and thermal properties.^3^ Graphene, with its single-layer structure and high electron mobility, is still attracting huge interest in both applied and fundamental research.^4,5^ Furthermore, the success of graphene has paved the way for the exploration of other two-dimensional materials, such as transition metal dichalcogenides (TMDs),^6,7^ hexagonal boron nitride (h-BN),^8,9^ black phosphorus,^10^ MXenes^11,12^ and topological insulators (TIs).^13,14^ These two-dimensional materials exhibit unique properties that can be tailored for various applications, ranging from electronics and optoelectronics to energy storage and catalysis.^15–17^ The interdisciplinary field of nanoscience, in combination with graphene and other two-dimensional materials, continues to unlock new possibilities for advanced materials and devices with enhanced performance and novel functionalities.^18,19^

Beyond that mentioned, two-dimensional materials based on group-IV have been intensively studied. Graphene and its 2D analogous, including silicene, germanene, and stanene, have gained significant research interest in the field of two-dimensional materials due to their unique physical-chemistry properties.^20^ All monoelemental group-IV 2D honeycomb materials beyond graphene (C), i.e., silicene (Si), germanene (Ge), stanene (Sn), and plumbene (Pb), have been experimentally obtained.^21–25^ Moreover, the combination of silicon and germanium in SiGe alloys allows for tunable bandgaps, enabling their use in various electronic and optoelectronic applications.^26^ Other combinations are also possible;^27^ thus the exploration and development of these Group-IV-based two-dimensional materials offer exciting opportunities for advancing nanoscience and expanding the functionalities of future devices.

Besides that, it is well-known that functionalization allows to improve or precisely modify the physical-chemistry properties of two-dimensional materials.^28–30^ In particular, the incorporation of fluorine atoms or fluorine-containing functional groups onto the surface of 2D materials can lead to significant changes in their electronic, optical, and chemical properties.^31^ Fluorine (F) has been extensively studied for its effects on 2D materials due to its strong electronegativity and unique chemical properties.^32^ One notable effect of fluorine is the modulation of the band structure and bandgap of 2D materials. Fluorine functionalization can induce bandgap opening or widening in materials that are otherwise semimetallic or have narrow bandgaps.^33^ This property has been explored in graphene, where fluorination can transform the material from a zero-gap semimetal to a semiconductor with a tunable bandgap.^34,35^ Furthermore, fluorination of Group-IV materials can modify their surface reactivity and interaction with other materials, leading to improved performance in catalysts, sensors, and energy storage devices.^36^ The controlled introduction of fluorine atoms onto the surface of Group-IV materials offers exciting opportunities for tailoring their properties and expanding their applications in areas such as electronics, sensors, catalysis, and energy conversion.^37^

In this context, the present work provides a comprehensive atomistic investigation of the hexagonal XYF_2_ family (X, Y = C, Si, Ge, Sn; X ≠ Y) using hybrid density-functional theory with spin–orbit coupling (SOC). In addition to the monolayers, we examined their corresponding van der Waals heterostructures in bilayer and trilayer configurations to assess the role of interlayer coupling on the electronic response. Dynamical and thermal stabilities are confirmed for all systems through phonon-dispersion calculations and *ab initio* molecular dynamics simulations (AIMD). Our results show that these fluorinated group-IV materials can exhibit either direct or indirect band gaps depending on composition and stacking mode, with pronounced band-edge reorganizations induced by interlayer hybridization. To evaluate their potential for photocatalytic water splitting, we determined the absolute band-edge positions relative to the vacuum level for all materials.

## Computational Methods

Ab initio calculations were performed for the XY and XYF_2_ 2D hexagonal materials (X, Y = C, Si, Ge, Sn; X ≠ Y) using density functional theory (DFT)^38,39^ implemented in the Vasp computational package.^40,41^ Since exchange-correlation functionals tend to underestimate energy gaps, we employed the GGA-PBE functional^42^ in conjunction with the hybrid functional described by Heyd-Scuseria-Ernzerhof (HSE06).^43,44^ For all electronic property calculations, the spin–orbital coupling (SOC) is considered. The ion-electron interaction was modeled using the projector augmented-wave potential (PAW),^45^ with valence electrons represented by a plane wave with a cutoff energy of 600 eV. The Brillouin zone was defined using a Γ-centered grid with dimensions of 12 × 12 × 1.^46^ The convergence parameters used for relaxation of atomic positions/lattice parameters and system energy were 1 × 10^–3^ eV/Å and 5 × 10^–8^ eV, respectively. Also, calculations with dipole corrections and van der Waals interactions (DFT-D3)^47,48^ were performed. To eliminate spurious interactions between periodic images, a vacuum of at least 20 Å was introduced along the *z*-axis. The dynamic and thermal stability of the XY and XYF_2_ systems were investigated through ab initio molecular dynamics simulations using the Nosé-Hoover thermal bath method^49^ and phonon dispersion calculations (DFPT) as implemented in the Phononpy computational package.^50^ In both cases, supercells with dimensions of 4 × 4 × 1 were employed. The elastic constants presented were determined using the Vaspkit computational package.^51^

## Results and Discussion

We begin our study by analyzing the structural properties of the hexagonal monolayers XY and XYF_2_. The crystal structure can regarded as two interleaving triangular lattices, where each atom in one sublattice X has 3 nearest neighbors in sublattice Y and vice versa. In Figure 1(a) we present a top view of the investigated materials. In Figure 1(b), we present a schematic representation of the first Brillouin zone. In Figure 1(c–e) we show side view of the hexagonal monolayers XY and XYF_2_. The systems (X = C; Y = Ge, Si, Sn) possess a flat hexagonal lattice [Figure 1(c)] The materials (X = Si, Ge; Y= Ge, Sn) have a buckling height (h) that increases as a function of the atomic ratio of the atoms in the material. We also verified that all fluorinated materials were buckled. The key structural parameters are summarized in Table 1.

**Figure 1:**
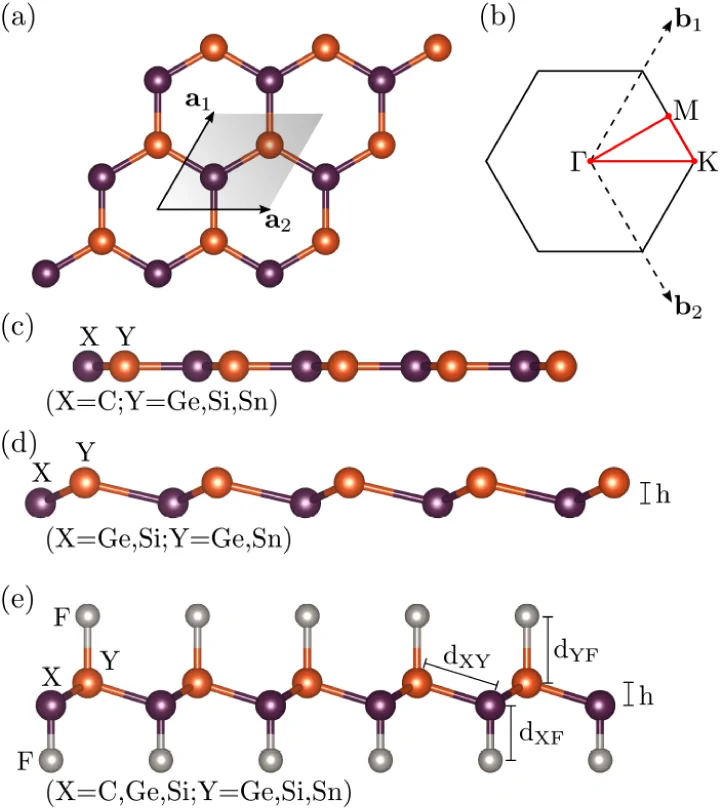
(a) Top view of the hexagonal monolayer XY and XYF_2_ materials. The unit cell is identified by the gray area. (b) Schematic view of the first Brillouin zone. The lattice vectors and the high-symmetry points are highlighted. (c–e) Side view of the investigated hexagonal monolayer materials.

**Table 1:** Lattice Parameters (a) and Bond Lengths (d_XY_, d_XF_ and d_YF_) and Bucking Height (h) for the Systems XY e XYF_2_ ^a^

System	a	d_XY_	h	System	a	d_XY_	d_XF_	d_YF_	h
CSi	3.07	1.77	^b^	CSiF_2_	3.13	1.90	1.42	1.59	0.56
CGe	3.22	1.86	^b^	CGeF_2_	3.29	1.99	1.41	1.73	0.58
CSn	3.55	2.05	^b^	CSnF_2_	3.63	2.19	1.42	1.90	0.62
SiGe	3.91	2.32	0.52	SiGeF_2_	4.03	2.42	1.61	1.76	0.65
SiSn	4.23	2.53	0.65	SiSnF_2_	4.39	2.62	1.62	1.92	0.67
GeSn	4.30	2.59	0.74	GeSnF_2_	4.49	2.66	1.77	1.92	0.60

a All structural parameters are presented in Å.

b <10^–3^.

The HSE06-DFT calculated lattice parameters of the XY as well as the XYF_2_ materials increase with the covalent radius of the XY elements, as shown by Table 1. For the hexagonal monolayers CSi, CGe and CSn the calculations clearly show the absence of a buckling height, thus the atoms in such materials are in a sp^2^ hybridization. The calculated buckling heights (h) for the pristine SiGe, SiSn and GeSn also increase from X, Y = Si, Ge, Sn (see Figure 1(d)), following a competition between sp^2^ + p and sp^3^ hybridization. These results are in good agreement with previous works.^52^ The same competition also governs the equilibrium geometry of the 2D fluorinated XYF_2_ materials. Moreover, the electronegativity difference between the X, Y, and F atoms plays a decisive role, and it increases systematically along the C → Sn series. Our calculations indicate that the degree of sp^3^ hybridization becomes more pronounced in SiSnF_2_ and weaker in CSiF_2_. Additional atomic relaxations further enlarge the XYF_2_ lattice constants as the system reduces the strain energy associated with fluorination.

The lattice parameters of the XY as well as the XYF_2_ materials increase with the covalent radius of the XY elements, as shown by Table 1. For the hexagonal monolayers CSi, CGe and CSn the calculations clearly show the absence of a buckling height, thus the atoms in such materials are in a sp^2^ hybridization. The calculated buckling heights (h) for the pristine SiGe, SiSn and GeSn also increase from X, Y = Si, Ge, Sn (see Figure 1(d)), following a competition between sp^2^ + p and sp^3^ hybridization. The same competition also governs the equilibrium geometry of the 2D fluorinated XYF_2_ materials. Moreover, the electronegativity difference between the X, Y, and F atoms plays a decisive role, and it increases systematically along the C → Sn series. Our calculations indicate that the degree of sp^3^ hybridization becomes more pronounced in SiSnF_2_ and weaker in CSiF_2_. Additional atomic relaxations further enlarge the XYF_2_ lattice constants as the system reduces the strain energy associated with fluorination.

The elastic constants are presented in Table 2. By examining the elastic constants of the materials, we find that C_11_ = C_22_ > 0, C_66_ = 1/2­(C_11_ – C_12_) > 0, and $C_{11}C_{22}-C_{12}^{2}>0$ , for all materials. Thus, according to the Born criteria, we can infer that the hexagonal monolayers XY and XYF_2_ materials are all mechanically stable.

**Table 2:** Calculated Elastic Constants (in Units of N/m) for All Investigated Hexagonal Monolayers XY and XYF_2_

**System**	C_11_	C_12_	**System**	C_11_	C_12_
CSi	181.40	55.38	CSiF_2_	121.20	22.08
CGe	157.43	51.21	CGeF_2_	92.61	16.93
CSn	114.37	45.79	CSnF_2_	63.56	15.17
SiGe	58.63	19.98	SiGeF_2_	44.56	8.82
SiSn	44.24	15.97	SiSnF_2_	32.91	7.93
GeSn	37.03	13.22	GeSnF_2_	23.68	8.01

We investigated the thermal and dynamical stability of the hexagonal XY and XYF_2_ monolayers by combining *ab initio* molecular dynamics (AIMD) simulations and phonon-spectrum analysis. Thermal stability was assessed through AIMD simulations performed at room temperature using a 1 fs time step and a Nosé–Hoover thermostat for simulation times up to 10 ps. The temporal evolution of the total potential energy, together with snapshots of the final atomic configurations shown in Figure 2(a1–a12), indicates that all monolayers retain their structural integrity throughout the simulations. Dynamical stability was further examined via phonon-dispersion calculations. As shown in Figure 2(b1–b12), all systems exhibit phonon spectra free of imaginary modes, demonstrating that the XY and XYF_2_ monolayers are dynamically stable as free-standing structures.

**Figure 2:**
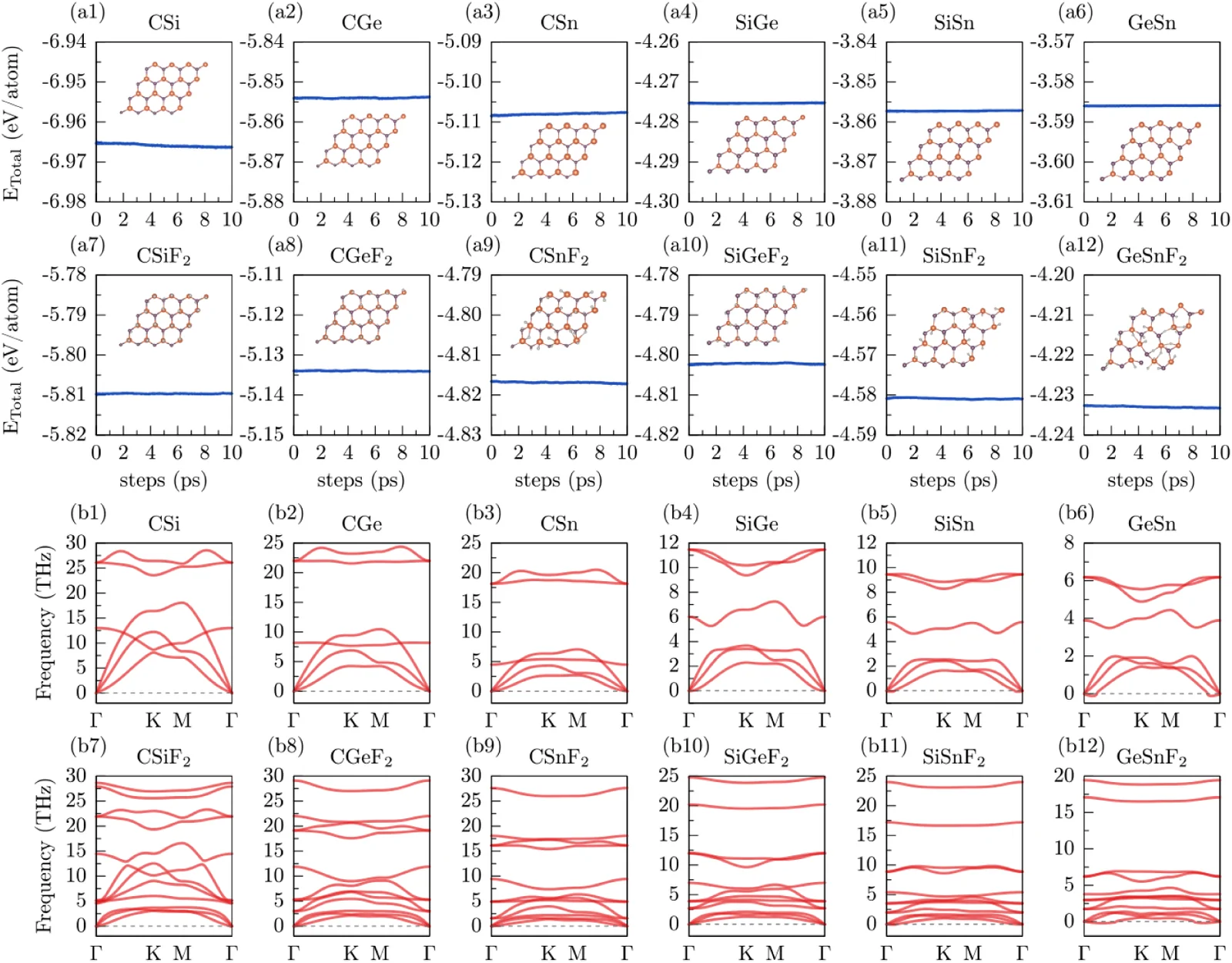
Total energy fluctuation along the AIMD simulations (at 300 K) of the hexagonal monolayer XY and XYF_2_ materials, with snapshots of the crystal structures at 10 ps (a1–a12); Phonon band structure of the monolayers XY and XYF_2_ (b1–b12).

In addition, the adsorption of fluorine atoms in XY (XY → XYF_2_) is an exothermic process. By comparing the total energies of the separated components, namely XY and an isolated F2 molecule, with the total energy of the final system, XYF_2_; we find an energy gain ranging from 2.75 eV (CGeF_2_) to 3.61 eV (SiSnF_2_) per F atom.

While our phonon and AIMD calculations confirm the dynamical and thermal stability of the final pristine and fluorinated XY monolayers as local minima, it is crucial to address their thermodynamic metastability and synthetic feasibility. Most of these heteronuclear 2D lattices are likely to reside above the convex hull compared to their 3D bulk counterparts. Furthermore, the substantial energy gain associated with F adsorption (ranging from 2.75 to 3.61 eV per F atom) highlights a significant experimental challenge. If fluorination is conducted using aggressive experimental methods, the massive local energy released during the X–F and Y–F bond formation could overcome the kinetic barriers preserving the 2D phase, causing the structure to collapse into a 3D thermodynamic minimum or amorphize. Consequently, the experimental realization of these XYF_2_ monolayers may benefit from kinetically controlled synthesis pathways. These could involve *soft* fluorination approaches, such as low-temperature XeF_2_ vapor exposure,^53,54^ together with the use of high-thermal-conductivity substrates (e.g., h-BN or metallic surfaces).^55^ Such substrates may serve as efficient heat sinks, facilitating the dissipation of the exothermic energy generated during fluorination and reducing the likelihood of structural degradation of the metastable 2D lattice.^56^

The electronic band structures calculated by HSE06 + SOC of the monolayers XY and XYF_2_ are shown in Figure 3. In the nonfluorinated case, the C-based systems (CSi and CSn) exhibit indirect semiconducting gaps, while CGe is a direct gap material. The heavier SiGe, SiSn and GeSn compounds display narrow gaps. Upon fluorination, the electronic landscape changes significantly: in CSi, CGe, and CSn the band structures reorganize while preserving wide-gap semiconducting behavior, whereas in SiGe and SiSn the small gaps broaden significantly, leading to well-defined semiconducting states. For GeSn, the bandgap remains narrow after fluorination, decreasing only slightly from 0.33 to 0.28 eV. This limited response to fluorination arises because GeSn already exhibits a strongly sp^3^-like bonding environment, and the relatively weak polarity of the Ge–F and Sn–F bonds produces only minor structural and electronic modifications. In addition, the band-edge states in GeSn are dominated by Ge-p and Sn-p orbitals, which are less sensitive to fluorine-induced rehybridization, while the substantial spin–orbit coupling associated with Sn further maintains the bands in close energetic proximity. As a consequence, fluorination induces only a small adjustment of the bandgap in GeSn.

**Figure 3:**
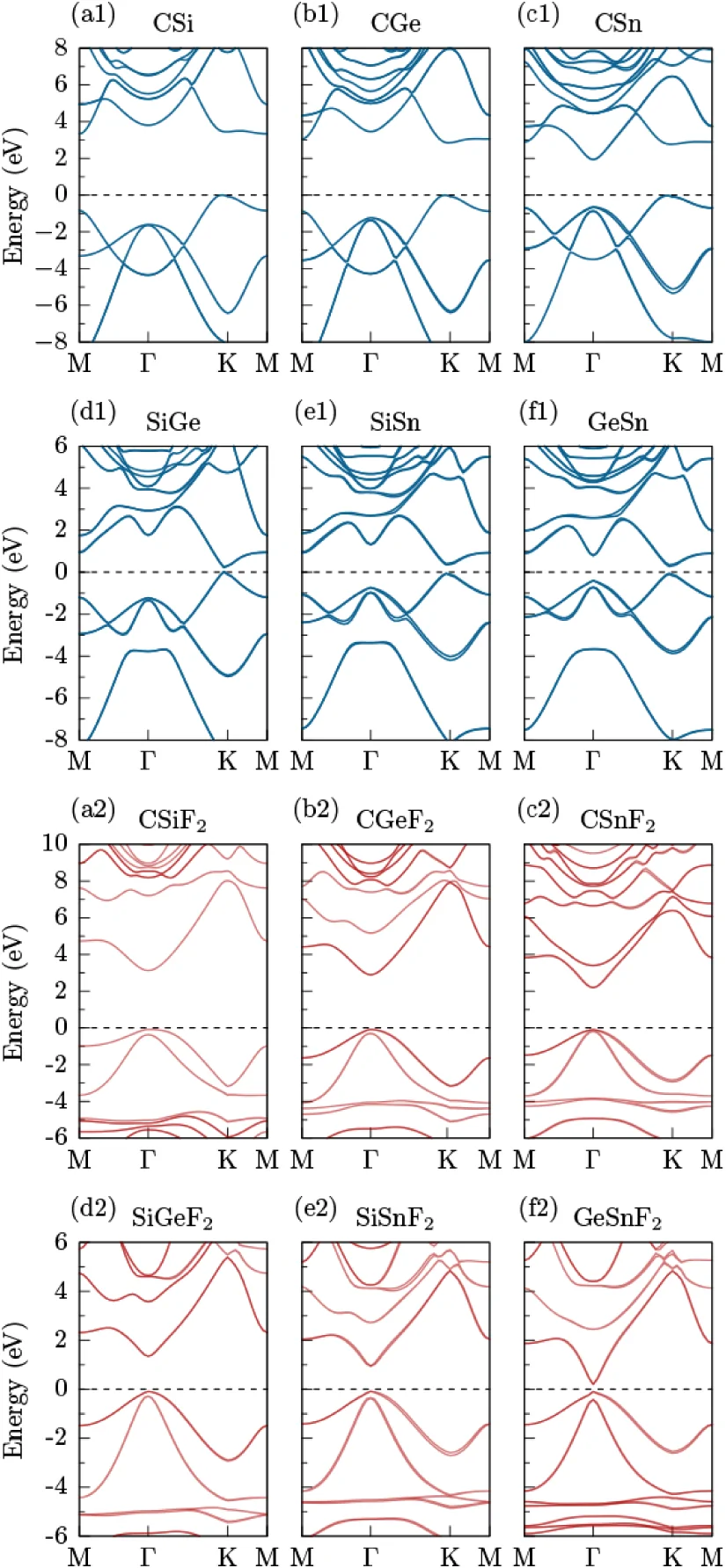
HSE06+SOC electronic band structures of XY (a1–f1) and XYF_2_ (a2–f2). All band structures were aligned with respect to the Fermi level (E-E_F_ = 0 eV).

The calculated bandgaps for both HSE06 and HSE06+SOC are summarized in Table 3. For the carbon-based systems (CSi, CGe, CSn), the bandgaps span a relatively wide range and include cases where the gap is indirect (indicated by asterisks). Fluorination generally increases the bandgap, most notably in SiGe, SiSn, and GeSn, reflecting the enhanced sp^3^ character and the stronger X/Y–F bonding introduced by the terminal fluorine atoms. The impact of spin–orbit coupling (SOC) is strongly dependent on the chemical composition of the system. For lighter-element compounds (e.g., C- and Si-based systems),^57–59^ SOC introduces only negligible changes in the bandgap and band-edge positions, reflecting the weak relativistic effects in these elements. In contrast, for Ge- and especially Sn-containing systems, SOC produces more pronounced band-edge shifts and bandgap renormalization due to stronger relativistic interactions.^60–62^ Although SOC does not qualitatively alter the electronic character of the systems, it remains important for an accurate description of band-edge positions. Band structures obtained from HSE06 calculations without SOC are shown in the Supporting Information (Figure S1).

**Table 3:** DFT-Calculated Bandgap (eV) for XY and XYF_2_ Systems

**System**	HSE	HSE + SOC	**System**	HSE	HSE + SOC
CSi	3.41^a^	3.28^a^	CSiF_2_	3.21	3.21
CGe	2.93	2.85	CGeF_2_	3.11	2.97
CSn	1.96^a^	1.96^a^	CSnF_2_	2.33	2.27
SiGe	0.19	0.19	SiGeF_2_	1.46	1.43
SiSn	0.39	0.41	SiSnF_2_	1.07	1.02
GeSn	0.34	0.33	GeSnF_2_	0.35	0.28

a Indirect bandgap.

Thus, a trend emerges (Table 3): lighter systems such as CSi and CGe reach the largest absolute postfluorination bandgaps, 3.21 and 2.97 eV, due to strong C–F and Si–F bonding. In contrast, Sn-rich systems, though ending with smaller absolute gaps, show the largest relative enhancements: CSn increases from 1.96 to 2.27 eV (+16%), SiGe from 0.19 to 1.43 eV (+653%), and SiSn from 0.41 to 1.02 eV (+149%). GeSn is exceptional, with a slight reduction from 0.33 to 0.28 eV (−15%), while CSi also shows a minor decrease (−2%). Taken together, the fluorinated systems span a wide gap range, from narrow bandgap GeSnF_2_ (0.28 eV) to wide-gap semiconductors such as CSiF_2_ (3.21 eV) and CGeF_2_ (2.97 eV). These reductions and enhancements can be rationalized: in CSi, fluorination perturbs an already wide gap, leading only to minor band alignment shifts, whereas in GeSn the strong spin–orbit interaction inherent to Sn counteracts the gap-opening tendency of fluorination, even narrowing the gap. Overall, fluorination stabilizes the lattice, reshapes the orbital character at the band edges, and, in most systems, significantly widens the bandgap by combining charge transfer with reduced orbital overlap.

Following the analysis of the electronic band structures (Figure 3) and the corresponding bandgaps (Table 3), the carrier dynamics can be further quantified through the evaluation of the effective masses. Since the band dispersion near the extrema directly controls charge transport, the effective mass (m*) of electrons and holes ( $m_{e}^{*}$ and $m_{h}^{*}$ , respectively) emerges as a key parameter governing carrier mobility, with direct implications for processes such as photocatalysis.

A clear correlation is observed between the curvature of the bands in Figure 3 and the effective masses summarized in Table 4. For the pristine XY monolayers [Figure 3(a1–f1)], a pronounced anisotropy is found. In CSi, the conduction band is highly dispersive along M−Γ, yielding a small electron mass (0.220 *m*_0_), whereas along M–K it becomes significantly flatter, leading to a much larger value (2.914 *m*_0_). A similar directional dependence is observed in CGe, with electron masses of 0.393 *m*_0_ (K−Γ) and 1.201 *m*_0_ (K–M). In CSn, the electron masses are nearly isotropic (0.249 and 0.265 *m*_0_ along Γ–M and Γ–K, respectively), indicating a more uniform band dispersion.

**Table 4:** Electron $\left(m_{e}^{*}\right)$ and Hole $\left(m_{h}^{*}\right)$ Effective Masses (in Units of *m*_0_) along High-Symmetry Directions for Pristine and Fluorinated XY Monolayers, Obtained from the Curvature of the Bands near the Extrema

System	m_*e*_		m_*h*_	
CSi^a^	2.914 (M–K)	0.220 (M−Γ)	–0.402 (K−Γ)	–0.695 (K–M)
CGe	0.393 (K−Γ)	1.201 (K–M)	–0.357 (K−Γ)	–0.613 (K–M)
CSn^a^	0.249 (Γ–M)	0.265 (Γ–K)	–0.356 (K−Γ)	–0.737 (K–M)
SiGe	0.102 (K−Γ)	0.700 (K–M)	–0.098 (K−Γ)	–0.468 (K–M)
SiSn	0.281 (K−Γ)	0.801 (K–M)	–0.278 (K−Γ)	–0.523 (K–M)
GeSn	0.300 (K−Γ)	0.866 (K–M)	–0.297 (K−Γ)	–0.550 (K–M)
CSiF_2_	0.479 (Γ–M)	0.479 (Γ–K)	–1.203 (Γ–M)	–1.184 (Γ–K)
CGeF_2_	0.335 (Γ–M)	0.335 (Γ–K)	–0.925 (Γ–M)	–0.902 (Γ–K)
CSnF_2_	0.206 (Γ–M)	0.206 (Γ–K)	–0.833 (Γ–M)	–0.842 (Γ–K)
SiGeF_2_	0.180 (Γ–M)	0.180 (Γ–K)	–0.601 (Γ–M)	–0.615 (Γ–K)
SiSnF_2_	0.124 (Γ–M)	0.124 (Γ–K)	–0.578 (Γ–M)	–0.562 (Γ–K)
GeSnF_2_	0.092 (Γ–M)	0.092 (Γ–K)	–0.566 (Γ–M)	–0.557 (Γ–K)

a Indirect bandgap.

In contrast, the heavier systems SiGe, SiSn, and GeSn exhibit relatively small electron effective masses along K−Γ, with values of 0.102, 0.281, and 0.300 *m*_0_, respectively, consistent with their more dispersive conduction bands near the band edges. The corresponding values along K–M (0.700, 0.801, and 0.866 *m*_0_) confirm the persistence of anisotropy. For all pristine systems, the hole effective masses are systematically larger in magnitude (e.g., −0.402 and −0.695 *m*_0_ in CSi), reflecting the comparatively flatter valence bands.

Upon fluorination [Figure 3(a2–f2)], the band extrema shift to the Γ point and the dispersion is reduced due to the transition from a planar sp^2^ to a buckled sp^3^-like structure. As a result, the electron effective masses become nearly isotropic, with identical values along Γ–M and Γ–K for each system (e.g., 0.479 *m*_0_ in CSiF_2_, 0.335 *m*_0_ in CGeF_2_, 0.206 *m*_0_ in CSnF_2_, and 0.092 *m*_0_ in GeSnF_2_). In contrast, the valence bands become significantly flatter, leading to larger hole effective masses, reaching −1.203 *m*_0_ in CSiF_2_. Across the fluorinated series, both electron and hole effective masses decrease systematically from CSiF_2_ to GeSnF_2_, indicating a gradual recovery of band dispersion with increasing atomic number. These trends provide a consistent picture of carrier transport in these systems and will be further discussed in connection with the electrostatic and band-alignment properties presented below.

The orbital-projected band structures provide further insights into the orbital composition, hybridization effects, and bandgap variations of pristine XY and fluorinated XYF_2_ monolayers, clarifying the nature of the states at the band edges and the mechanisms underlying the fluorination-induced changes. Given the close similarity of the band structures within each family, we perform the orbital-resolved analysis only for a representative member. In the pristine systems (Figure 4), the valence band maximum (VBM) and conduction band minimum (CBM) are predominantly composed of p_*z*_ orbitals from the heavier group-IV elements (Ge, Sn), with notable p_*x*_/p_*y*_ admixture from the lighter partner atom (C or Si). This reflects in a strong sp^2^-like hybridization within the hexagonal lattice, while s-orbitals remain confined to deeper valence energies (below −6 eV). CSi (a1) presents an indirect bandgap of 3.28 eV, where the conduction-band edge is mainly derived from Si-3p_*z*_ orbitals and the valence-band edge from C-2p_*z*_. CGe (b1) shows a slightly smaller indirect gap of 2.85 eV, while CSn (c1) narrows further to 1.96 eV with flatter band dispersion. In contrast, SiGe (d1) exhibits an ultranarrow gap of 0.19 eV, SiSn (e1) opens to 0.41 eV, and GeSn (f1) remains a small-gap semiconductor with a band gap of 0.33 eV.

**Figure 4:**
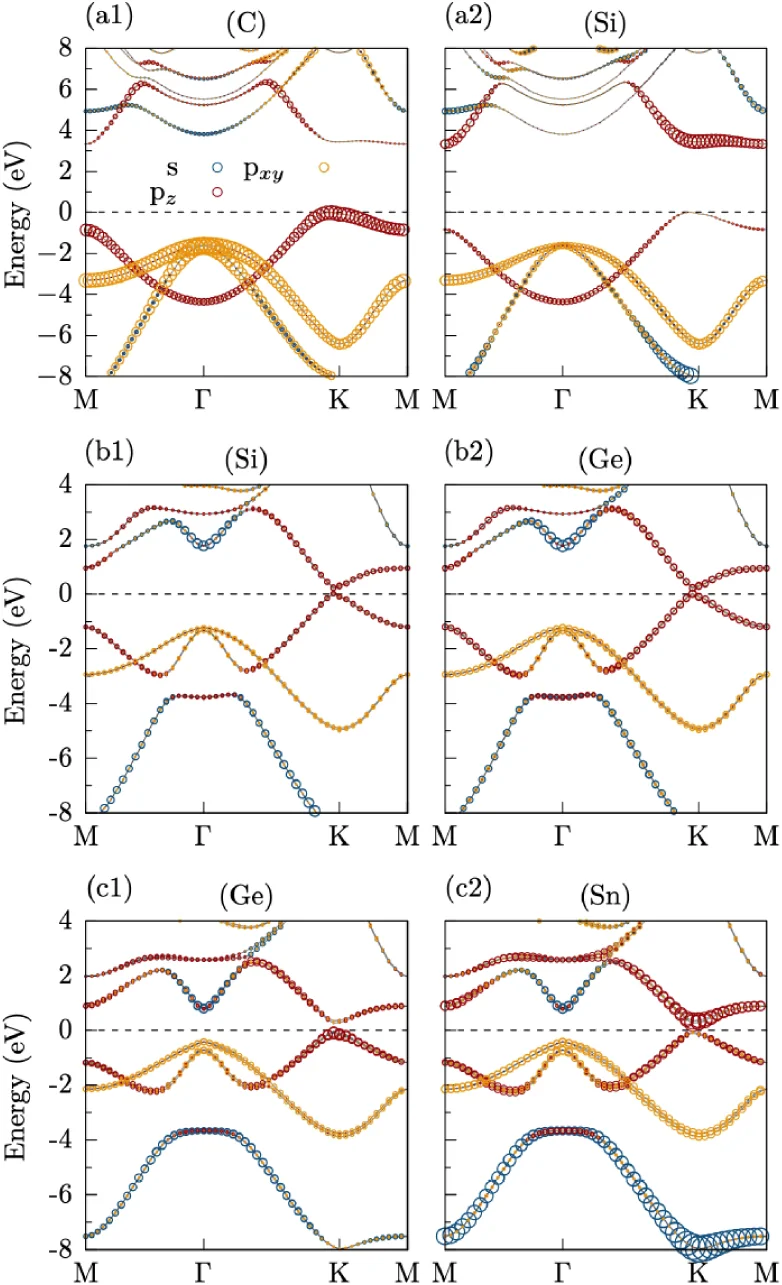
HSE06+SOC Projected Band Structure in s (blue), p*_xy_* (red), and p*_z_* (orange) orbitals for XY materials (a1–c2), with energy bands aligned to the Fermi level set to zero (E_F_ = 0 eV).

In contrast to the pristine systems discussed in Figure 4, VBM and CBM are governed by delocalized p_*z*_ states characteristic of an sp^2^-like framework, the projected band structures of the fluorinated monolayers shown in Figure 5 reveal how fluorination fundamentally reorganizes the orbital character near the gap. Overall, fluorination induces a dual modification of the electronic structure: (i) electronic localization, driven by the strong electronegativity of fluorine, which withdraws charge from the host atoms, suppresses π–π* overlap, and weakens the dispersive p_*z*_ lattice; and (ii) structural rehybridization, as F adsorption promotes an out-of-plane distortion that converts the planar sp^2^ lattice into a buckled sp^3^-like geometry. As a consequence, the edge states dominated by p_*z*_ of the pristine monolayers are progressively replaced by mixed contributions off F-p_*x*_/p_*y*_–F-p, leading to flatter bands and a reorganization of the valence and conduction-edge positions. Such fluorination-driven structural changes govern the band gap behavior, with VBM and CBM shifts dictated by p_*z*_ suppression and X–F bond strength.

**Figure 5:**
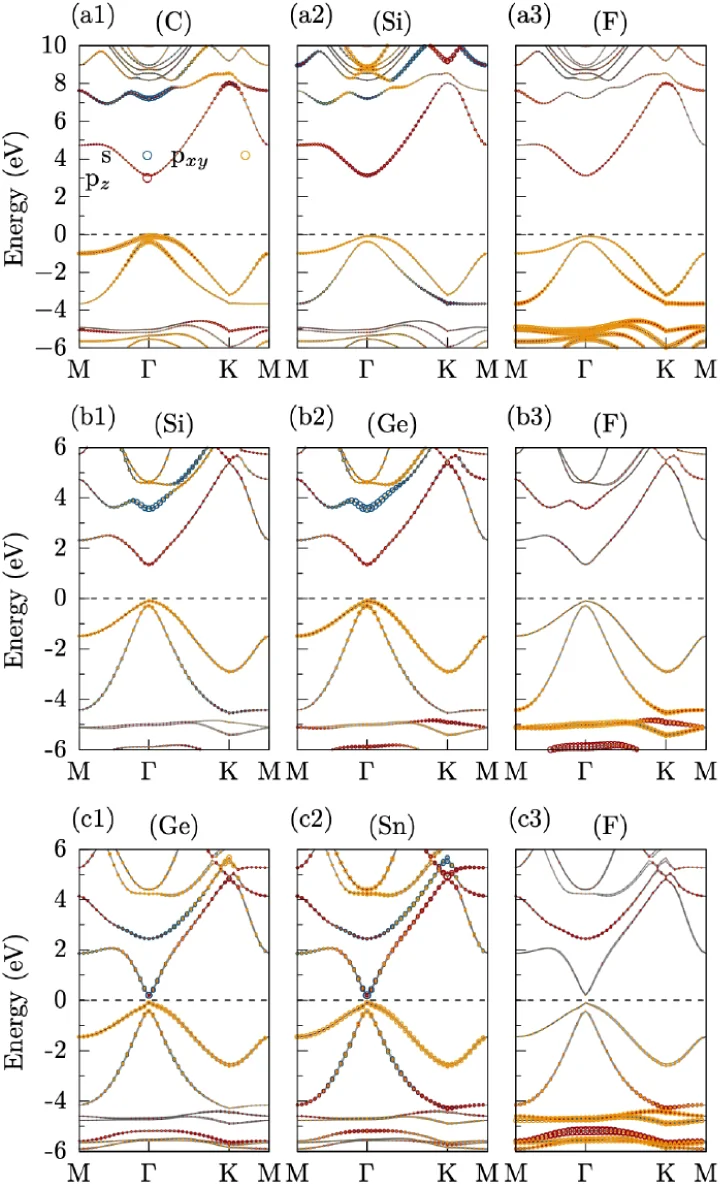
HSE06+SOC Projected Band Structure in s (blue), p*_xy_* (red), and p*_z_* (orange) orbitals for hexagonal monolayers XYF_2_ materials (a1–c3). The energy bands were aligned to the vacuum level.

To understand the nonmonotonic band gap evolution upon fluorination, it is essential to consider the charge distribution among the valence orbitals and the electric dipole moments of the pristine and fluorinated monolayers, as summarized in Table 5. While lattice puckering typically promotes gap opening via purely covalent sp^3^ rehybridization, the high electronegativity of fluorine introduces a complex competition that cannot be explained solely by a simple monotonic dependence on the sp^3^ character.

**Table 5:** Calculated Absolute Electric Dipole Moments (|*μ*|) and Orbital Charge Populations for Hexagonal XY and XYF_2_ Monolayers (X, Y = C, Ge, Si, Sn; X ≠ Y)^a^

		*q*_*X*_			*q*_*Y*_			*q*_*X*F_			*q*_*Y*F_		
System	|μ| (D)	s	p	d	s	p	d	s	p	d	s	p	d
CSi	0.000	0.850	1.715	0.000	0.754	1.006	0.000	–	–	–	–	–	–
CGe	0.000	0.836	1.599	0.000	1.098	1.432	0.205	–	–	–	–	–	–
CSn	0.000	0.842	1.504	0.000	1.320	2.032	0.391	–	–	–	–	–	–
SiGe	0.024	0.803	0.877	0.000	1.159	1.312	0.060	–	–	–	–	–	–
SiSn	0.169	0.825	0.838	0.000	1.405	1.802	0.130	–	–	–	–	–	–
GeSn	0.133	1.207	1.154	0.034	1.429	1.683	0.114	–	–	–	–	–	–
CSiF_2_	0.007	0.833	1.626	0.000	0.729	0.981	0.000	1.755	4.356	0.000	1.753	4.424	0.000
CGeF_2_	0.401	0.832	1.530	0.000	1.067	1.345	0.258	1.754	4.369	0.000	1.754	4.356	0.000
CSnF_2_	0.701	0.840	1.463	0.000	1.280	1.934	0.480	1.753	4.369	0.000	1.751	4.348	0.000
SiGeF_2_	0.493	0.761	0.893	0.000	1.104	1.305	0.151	1.752	4.414	0.000	1.752	4.353	0.000
SiSnF_2_	0.804	0.772	0.863	0.000	1.332	1.855	0.295	1.751	4.412	0.000	1.750	4.347	0.000
GeSnF_2_	0.303	1.142	1.177	0.123	1.355	1.746	0.281	1.752	4.355	0.000	1.750	4.348	0.000

a Dipole moments are reported in Debye (D).

Due to its high electronegativity, fluorine strongly attracts electrons from both the X and Y atoms. The orbital occupations reveal a clear depletion of the s and p states on the Group-IV atoms upon fluorination. For heavier atoms such as Ge and Sn, this substantial electron withdrawal reduces the electronic screening of the nucleus, thereby increasing the effective nuclear charge (*Z*_eff_). Consequently, the initially high-energy d-orbitals are lowered in energy and hybridize with the remaining valence states. As shown in Table 5, the d-orbital occupation increases significantly after fluorination. For instance, in GeSnF_2_, the Ge d-electron population increases from 0.034 to 0.123 *e*, and the Sn d-electron population from 0.114 to 0.281 *e*. The participation of these extended d-states broadens the conduction band minimum (CBM), effectively pushing it downward. This mechanism suppresses the purely covalent sp^3^ character, resulting in a smaller band gap and directly counteracting the structural gap-opening tendency.

Furthermore, the band gap trends are strongly modulated by local bond polarization. For lighter, tightly bound systems such as CSi, the macroscopic dipole moment remains nearly zero (0.007 D) after fluorination, preserving macroscopic symmetry. However, the local electrostatic fields are drastically altered. The heavy transfer of charge to the F p-orbitals (∼4.3–4.4 *e*) creates highly ionic X–F and Y–F bonds. This strong local ionicity diminishes the intralayer covalent overlap integral between the X and Y atoms within the basal plane. As a result, the bonding–antibonding energy splitting is reduced compared to a purely covalent sp^3^ framework, which explains why CSi does not exhibit the anticipated large band gap enhancement. Conversely, for other XY combinations with pronounced electronegativity asymmetry, large net dipole moments emerge (e.g., 0.804 D for SiSnF_2_), introducing an internal Stark effect that further modulates the band edges.

Therefore, the assumption that a stronger sp^3^ character straightforwardly correlates with a larger band gap holds primarily for purely covalent frameworks. In these fluorinated monolayers, the band gap is ultimately dictated by a delicate interplay: the gap-opening tendency of structural puckering is counterbalanced by the loss of intralayer covalent overlap due to F-induced ionicity and the downward shift of d-orbital energies.

It is worth noting that the XYF_2_ structures investigated in this work represent the upper stoichiometric limit of full fluorination. Under realistic experimental conditions, the degree of functionalization is often limited, leading to partial or single-sided coverage rather than full saturation. To address this scenario, we have additionally simulated a less fluorinated model as a proof-of-concept, by constructing a 2 × 2 supercell of the SiGe monolayer with single-sided fluorine adsorption. The SiGe system was selected as a representative case because it exhibits one of the strongest responses to fluorination among the studied compounds. As detailed in the Supporting Information (Figure S2), reducing the fluorine concentration prevents the homogeneous transition into a fully saturated sp^3^ lattice. This partial functionalization yields a distinct electronic profile by modifying the valence and conduction band edges, offering narrow energy bandgaps and an insulator-to-metal transition that contrast sharply with the conventional semiconducting nature of the fully fluorinated SiGeF_2_ counterpart. This contrast underscores that controlling the degree of coverage provides a versatile strategy to experimentally tailor the electronic and spin-dependent properties of heteronuclear group-IV monolayers.

Following this brief assessment of partial functionalization, we return our focus to the fully saturated XYF_2_ monolayers. To further understand how these uniform structural modifications impact the surface properties and charge distribution of these pristine and fully functionalized systems, we shift our focus to the electrostatic behavior. Figure 6 presents the planar-averaged electrostatic potential for SiSn and SiSnF_2_ along the direction perpendicular to the plane (*ẑ*). The plateaus observed on both sides of the slab correspond to the vacuum energy level. By comparing these vacuum levels, it is possible to assess the presence of intrinsic asymmetry in the system, which may arise from differences in chemical composition, electronegativity, or structural distortions within the hexagonal monolayer. For CSi, CGe, and CSn, no asymmetry is observed (ΔΦ = 0.0 eV), whereas SiGe, SiSn, and GeSn exhibit only small potential differences (ΔΦ = 0.07, 0.41, and 0.31 eV, respectively). In contrast, fluorine functionalization, due to its high electronegativity, induces a significant asymmetry in all systems, as summarized in Table 6. For instance, in SiSnF_2_ we obtain ΔΦ = 1.81 eV [Figure 6(b)]. From the vacuum level and the corresponding energies of the valence-band maximum (VBM) and conduction-band minimum (CBM), one can determine the ionization energy (IE) and electron affinity (EA), as illustrated in Figure 6(c). In systems exhibiting a finite ΔΦ, the presence of an intrinsic dipole leads to side-dependent values of IE and EA. Such internal electric fields, as previously reported,^63–65^ play a central role in 2D photocatalytic platforms, particularly when combined with the spatial separation of VBM and CBM across opposite sides of the layer, promoting charge separation and reducing electron–hole recombination.

**Figure 6:**
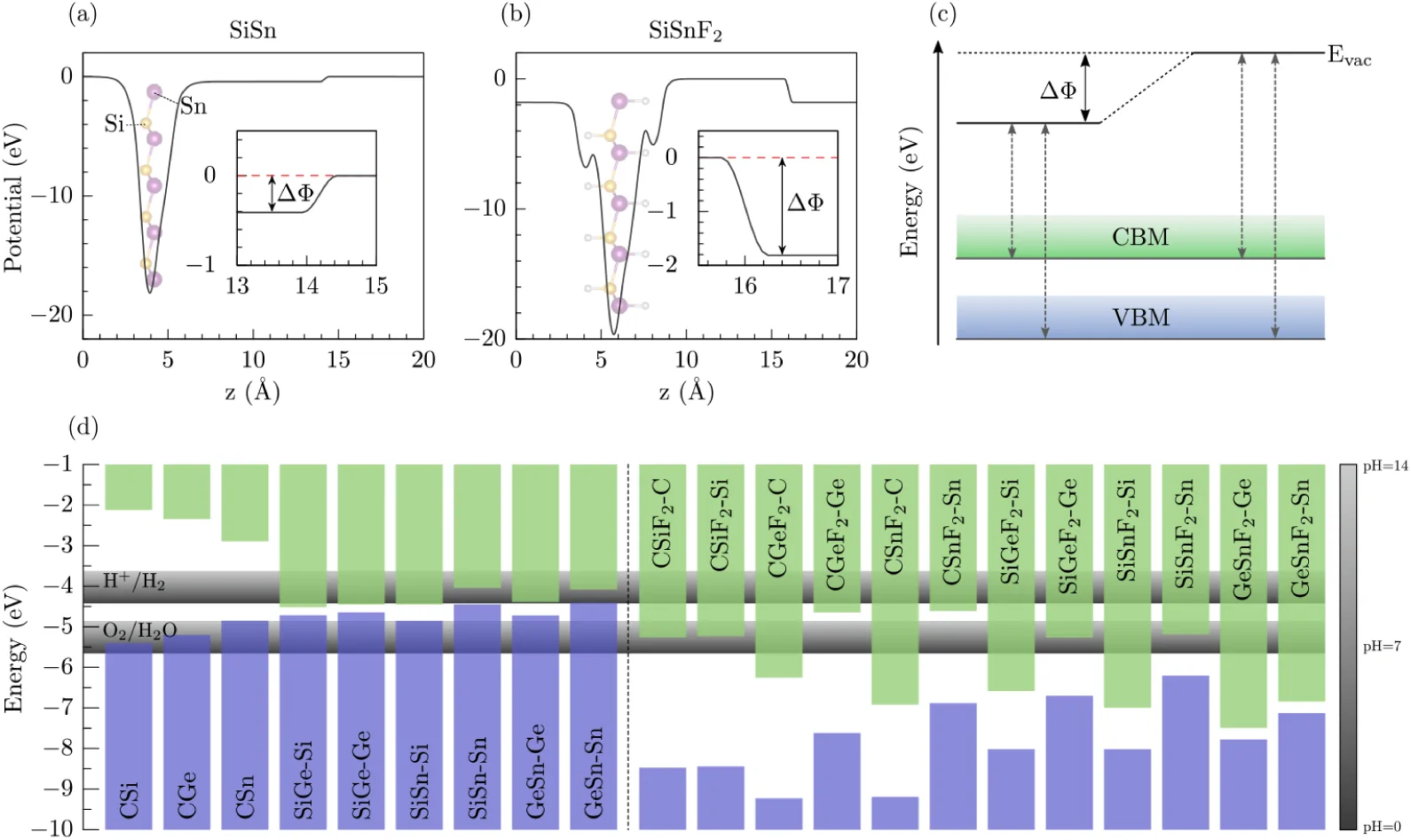
Potential along the *z* axis for some selected 2D materials: (a) SiSn and (b) SiSnF_2_. The insets show the potential difference (ΔΦ) in nonplanar materials. (c) Schematic illustration of the band offset for ΔΦ ≠ 0 materials, presenting the CBM (green) and VBM (blue) aligned with different vacuum levels of the system. (d) Band-edge alignments with respect to the vacuum level for all water-splitting candidate materials. We also show the reduction/oxidation potential of water with respect to the pH value (grayscale gradient region).

**Table 6:** Difference between the Vacuum Potentials (ΔΦ) for the Hexagonal Monolayers XY and XYF_2_ (X, Y = C, Ge, Si, Sn; X ≠ Y)

System	ΔΦ	System	ΔΦ
CSi	0	CSiF_2_	0.03
CGe	0	CGeF_2_	1.61
CSn	0	CSnF_2_	2.31
SiGe	0.07	SiGeF_2_	1.32
SiSn	0.41	SiSnF_2_	1.81
GeSn	0.31	GeSnF_2_	0.65

To evaluate the photocatalytic activity and band-edge positions of the XY and XYF_2_ 2D hexagonal materials (X = C, Si, Ge; Y = Si, Ge, Sn), Figure 6(d) shows the band alignment relative to the vacuum level, compared with the redox potentials of water over a pH range of 0–14. Although all materials exhibit direct band gaps, CSi, CGe, CSn, and all fluorinated XYF_2_ compounds do not satisfy the energetic requirements for water splitting. In these cases, fluorination strongly modifies the electronic structure, lowering the conduction-band minimum below the H^+^/H_2_ reduction potential and/or shifting the valence-band maximum above the O_2_/H_2_O oxidation level. In contrast, the remaining systems exhibit band-edge positions compatible with both redox potentials, enabling water splitting across the full pH range. Furthermore, as highlighted by Ji et al.,^66^ the intrinsic electric field in these structures promotes spatial separation of electrons and holes across different surfaces, which can reduce exciton recombination. It is important to note that our assessment of photocatalytic performance is based on band-edge alignment and electrostatic descriptors, which are widely used as first-order screening criteria. A quantitative evaluation of charge-carrier mobility and dynamics would require more advanced approaches (e.g., electron–phonon coupling or transport calculations) and is beyond the scope of the present study. Carrier transport in these systems is expected to correlate with the band dispersion near the band edges, where more dispersive bands indicate lower effective masses and potentially higher mobility.

The effective-mass analysis presented above provides additional support to this picture. Systems with more dispersive conduction bands tend to exhibit smaller electron effective masses, suggesting enhanced carrier mobility, whereas flatter valence bands lead to larger hole masses, indicating increased localization. Although a quantitative description of carrier dynamics is beyond the scope of the present work, these trends are consistent with the expected role of band dispersion in charge transport. In this context, the coexistence of favorable band-edge alignment, intrinsic electric fields, and effective-mass trends suggests that selected systems may benefit from improved charge separation and reduced recombination, which are key factors for photocatalytic performance.

In order to further discuss the properties of those materials we constructed, for 2D hexagonal monolayers with a closer lattice parameter [see Table 1], van der Waals (vdW) heterostructures. Figure 7 presents the atomic structures and corresponding electronic band structures of different heterobilayer systems. The electronic band structures of the three vdW heterostructure reveal distinct low-energy behaviors shaped by fluorination and layer composition. In the case of CSi+CSiF [Figure 7 (a)], the system retains a narrow direct bandgap of about 0.1 eV at Γ, indicating semiconductor character close to a semimetallic limit. Such a small gap suggests that thermal excitation at room temperature could readily activate carriers, while still preserving optical activity associated with direct transitions. By contrast, both SiSn+SiSnF_2_ [Figure 7 (b)] and GeSn+SiSnF_2_ [Figure 7 (c)] exhibit semimetallic dispersions, where valence and conduction bands overlap near the Fermi level. The orbital-projected markers, resolved into contributions from the top and bottom layers, highlight how the band edges are distributed across the heterostructure. In CSi+CSiF_2_, the valence and conduction states at Γ are strongly localized on opposite layers, helping stabilize the residual direct gap. In contrast, in the Sn-based bilayers, significant hybridization between top and bottom layers mixes the states near the Fermi level, producing overlapping dispersions and reinforcing their semimetallic character.

**Figure 7:**
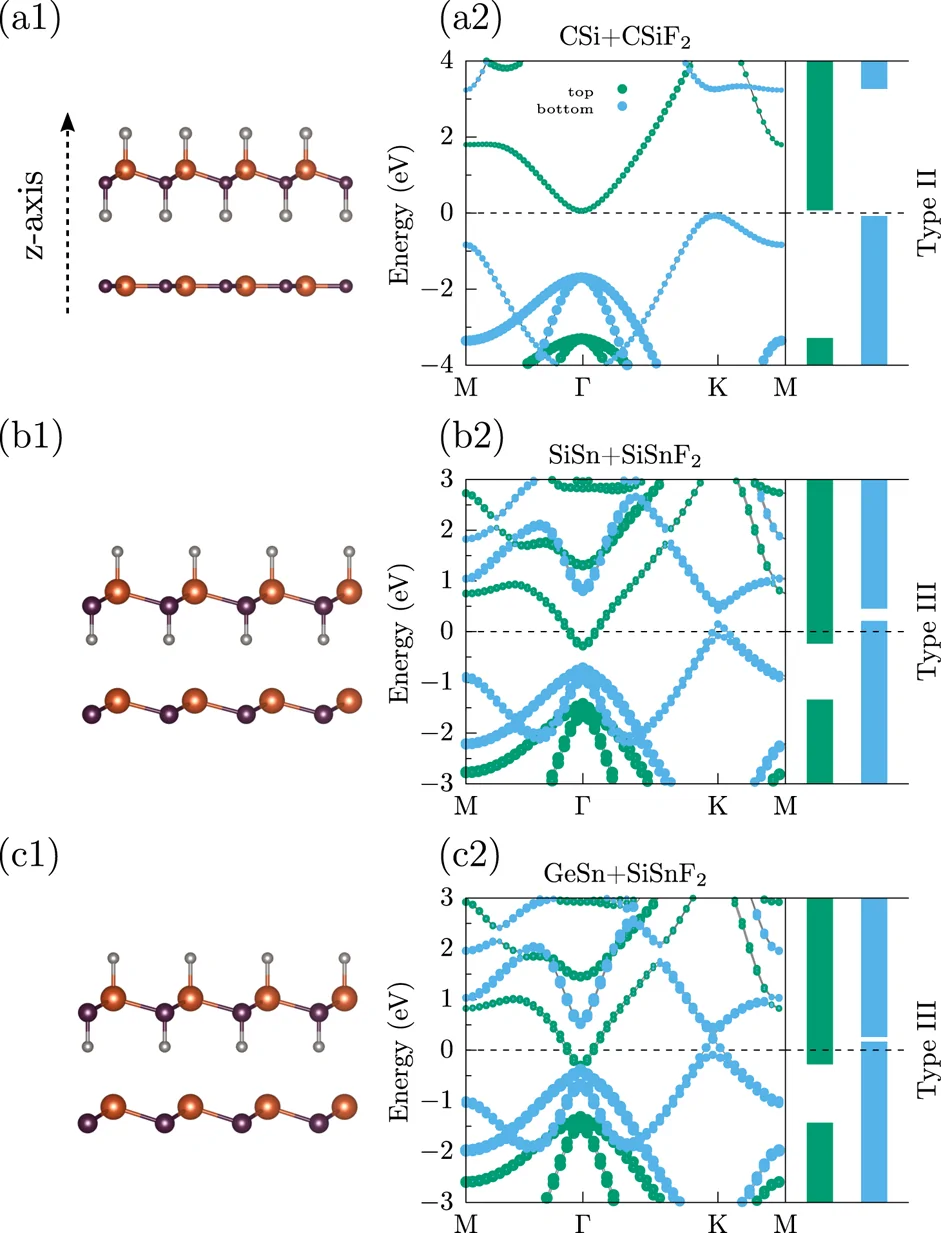
Atomic arrangements, layer-resolved electronic band structures and possible band alignments of: (a1, a2) CSi + CSiF_2_, (b1, b2) SiSn + SiSnF_2_ - and (c1, c2) GeSn + SiSnF_2_. All band structures were aligned with respect to the Fermi level (E-E_F_ = 0 eV).

Figure 7 presents the optimized geometries and layer-resolved band structures of the CSi+CSiF_2_, SiSn+SiSnF_2_, and GeSn+SiSnF_2_ heterobilayers, and illustrates how fluorination and chemical composition determine the interlayer band alignment. In CSi+CSiF_2_, the Figure 7(a) shows a clear type-II staggered configuration, where the conduction-band minimum and valence-band maximum occupy different monolayers. This separation is evident from the layer-projected states and reflects a charge-transfer regime dominated by spatially separated carriers, which stabilizes interlayer excitons and reduces direct electron–hole recombination. In contrast, the band structures of SiSn+SiSnF_2_ and GeSn+SiSnF_2_ displayed in the Figure 7(b, c) reveal a type-III (broken-gap) alignment, marked by the energetic overlap between valence-band states of one layer and conduction-band states of the other. This broken-gap arrangement eliminates the interlayer barrier highlighted in the band projections and favors band-to-band tunneling as the primary charge-transfer pathway. The larger overlap in GeSn+SiSnF_2_ showed that the stronger mixing of the projected states indicates a more efficient tunneling channel. Overall, the figure captures two distinct materials-physics regimes: type-II, governed by long-range charge separation, and type-III, governed by enhanced interlayer hybridization and quantum tunneling.

Figure 8 shows the HSE06 orbital-resolved electronic band structure of the vdW heterobilayers, allowing direct identification of the orbital origin of the electronic states near the Fermi level. Figure 8(a1–a3) presents results for the CSi + CSiF_2_ system, where the band edges are dominated by p_*z*_ orbitals from C and Si, with additional contributions from F orbitals, giving rise to the narrow gap of about 0.1 eV observed previously. In contrast, Figure 8(b1–b3) for SiSn+SiSnF_2_ and Figure 8(c1–c3) for GeSn+SiSnF_2_ reveal that the states around the Fermi level are strongly hybridized between Sn/Ge and Si p-orbitals together with F, leading to overlapping conduction and valence bands and hence to semimetallic behavior. In general, this analysis confirms that the opening of the gap in CSi+CSiF_2_ originates from a partial separation of the orbital contributions between the top and bottom layers, while enhanced interlayer orbital mixing in the heterobilayers containing Sn and Ge drives the transition to a semimetallic phase.

**Figure 8:**
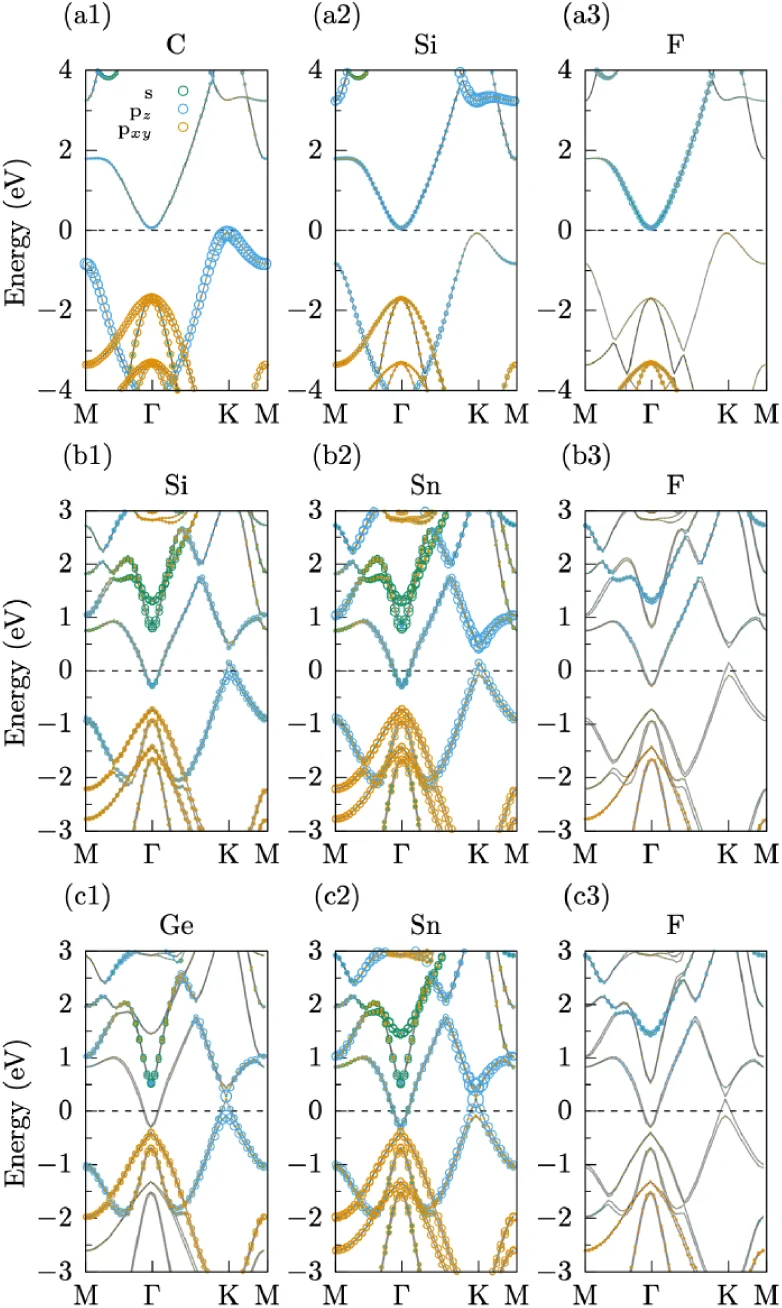
Orbital-resolved electronic band structures of: CSi+CSiF_2_ (a), SiSn+SiSnF_2_ (b) and GeSn+SiSnF_2_ (c). All band structures were aligned with respect to the Fermi level. Zero energy is set to be the Fermi level.

Figure 9 summarizes the structural and electronic evolution of CSi trilayers under distinct fluorination patterns. In Figure 9(a), only the middle layer is fully fluorinated, leaving the two outer layers in an sp^2^-bonded environment. Consequently, the trilayer retains the graphitic character, with π- and π*-derived states that remain strongly layer-polarized, as evidenced by the orbital-resolved bands. In this conformation, the materials exhibits a quite small indirect (K-Γ) bandgap of ∼0.022 eV. Figure 9(b), both outer layers are fully fluorinated, which enhance local σ bonding but still preserves the overall sp^2^ framework because the central layer remains unfluorinated. As a result, the π manifold is only moderately perturbed, and the electronic states continue to exhibit limited intersublayer hybridization. A tiny indirect (K-Γ) bandgap, about 0.017 eV, is characteristic of the material in this atomic arrangement. In contrast, Figure 9(c) corresponds to a configuration in which the outer layers are only partially fluorinated, a condition that substantially destabilizes the sp^2^ crystal lattice. Incomplete passivation produces strong local distortions and drives a fluorine-induced diamondization process, in which the lattice reconstructs into a buckled sp^3^-hybridized structure. This transition suppresses the π manifold, opens a sizable indirect (Γ-M) band gap of ∼1.94 eV, and yields σ-dominated dispersions throughout the Brillouin zone. The orbital-resolved bands for the diamondized structure display nearly uniform contributions from all three layers, demonstrating enhanced interlayer hybridization and the formation of an electronically symmetric, diamond-like covalent lattice within the partially fluorinated CSi trilayer.

**Figure 9:**
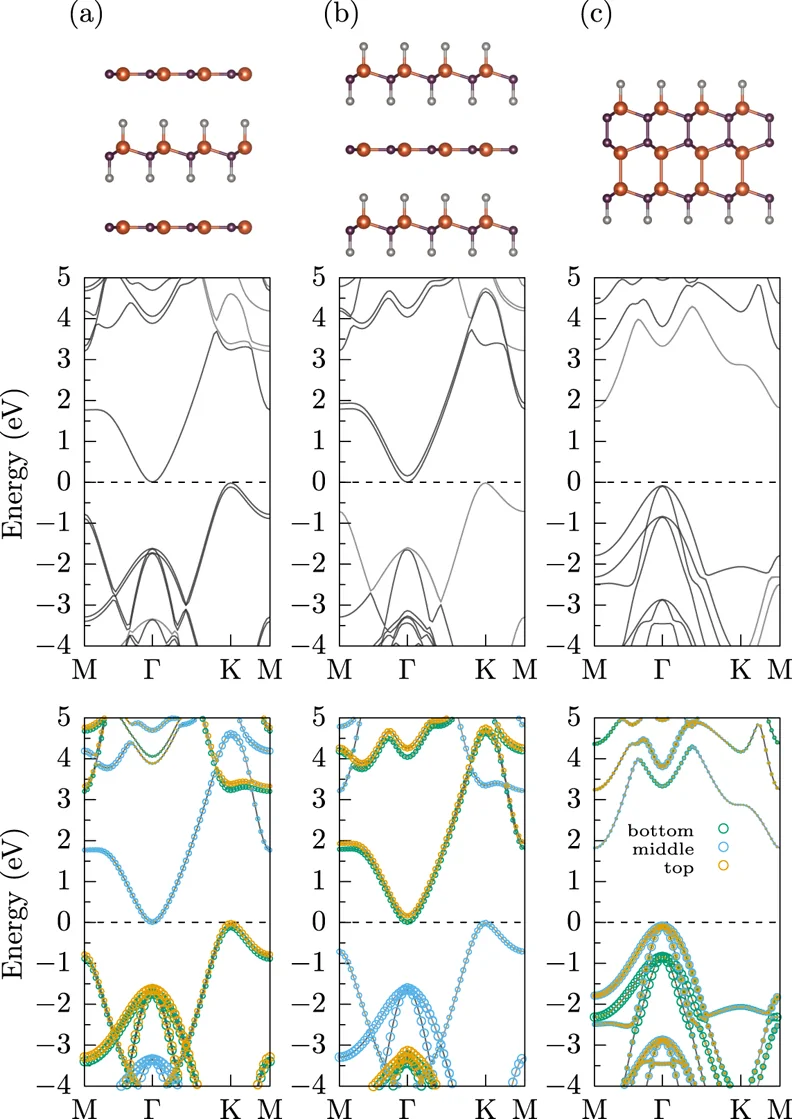
(a–c) Crystalline lattices, electronic band structures, and layer-resolved band structures. All band structures are aligned relative to the Fermi level (E_F_ = 0 eV).

## Conclusions

In this work, we carried out a systematic first-principles investigation of a family of hexagonal XY and fluorinated XYF_2_ monolayers (X, Y = C, Si, Ge, Sn; X ≠ Y). Our approach combines hybrid *ab initio* electronic-structure calculations, including spin–orbit coupling, with phonon-spectrum analyses and *ab initio* molecular-dynamics simulations. The results reveal a clear and systematic response of the structural and electronic properties to fluorination. All monolayers, pristine and functionalized, are dynamically and thermally stable, and the fluorination process is exothermic for every composition. Functionalization changes the planar sp^2^ lattices of the XY compounds into buckled sp^3^ structures, with the magnitude of buckling and lattice expansion increasing along the C → Sn chemical sequence. This structural rearrangement strongly affects the electronic landscape: while wide-gap systems such as CSi, CGe, and CSn undergoes only small gap modifications upon fluorination, narrow-gap compounds undergo substantial bandgap opening. In particular, SiGe increases from 0.19 to 1.43 eV and SiSn from 0.41 to 1.02 eV, demonstrating the strong influence of fluorine on hybridization and orbital ordering. GeSn remains nearly insensitive to functionalization, retaining its narrow-gap character.

The incorporation of fluorine atoms in the crystal lattices also introduces significant modifications in vdW heterostructures. By aligning the band edges of the bilayers to the vacuum level, we identify both type-II and type-III band offsets. Additionally, CSi-based trilayers undergo a complete transition from a graphitic sp^2^ lattice to a diamondized sp^3^ configuration, triggered by partial fluorination of the outer layers, resulting in substantial modifications of the electronic band structure.

Overall, these findings demonstrate that fluorination acts as a versatile and chemically robust mechanism for tailoring lattice stability, hybridization character, bandgap magnitude, and band-edge alignment in Group-IV two-dimensional systems, offering promising pathways for optoelectronic and photocatalytic applications.

## Supplementary Material



## Data Availability

GitHub Repository: https://github.com/dkuritza/papers/tree/main/2026/fluorination-effects.

## Notes

HSE06 and HSE06+SOC electronic band structures of the XY and XYF_2_ systems; electronic band structures of the partially fluorinated SiGe monolayer (PDF)
