# Supplementary material for: Fluorination Effects in Hexagonal XY (X, Y = C, Ge, Si, Sn; X ≠ Y) Monolayers and vdW Heterostructures: Insights from Ab Initio Calculations
Source: ACS Omega. 2026 Sep 1;11(38):57004–15. doi: 10.1021/acsomega.6c04011 (PMC13629059; doi:10.1021/acsomega.6c04011)
Supplement: Supplementary file 1 [file ao6c04011_si_001.pdf]

Supplementary Material

Fluorination Effects in Hexagonal XY (X,Y= C, Ge, Si, Sn; X≠Y) Monolayers and vdW Heterostructures: insights from *ab initio* calculations

Renato B. Pontes,[1,*] Danilo P. Kuritza,[2,3,†] Rafael Barbosa,[3,‡] and José E. Padilha[2,3,§]

[1]*Instituto de Física, Universidade Federal de Goiás, Campus Samambaia, 74690-900, Goiânia, GO, Brazil*

[2]*Campus Avançado Jandaia do Sul, Universidade Federal do Paraná, 86900-000, Jandaia do Sul, PR, Brazil.*

[3]*Departamento de Física, Universidade Estadual de Maringá, Maringá 87020-900, PR, Brazil*

(Dated: August 27, 2026)

HSE AND HSE+SOC BAND STRUCTURES

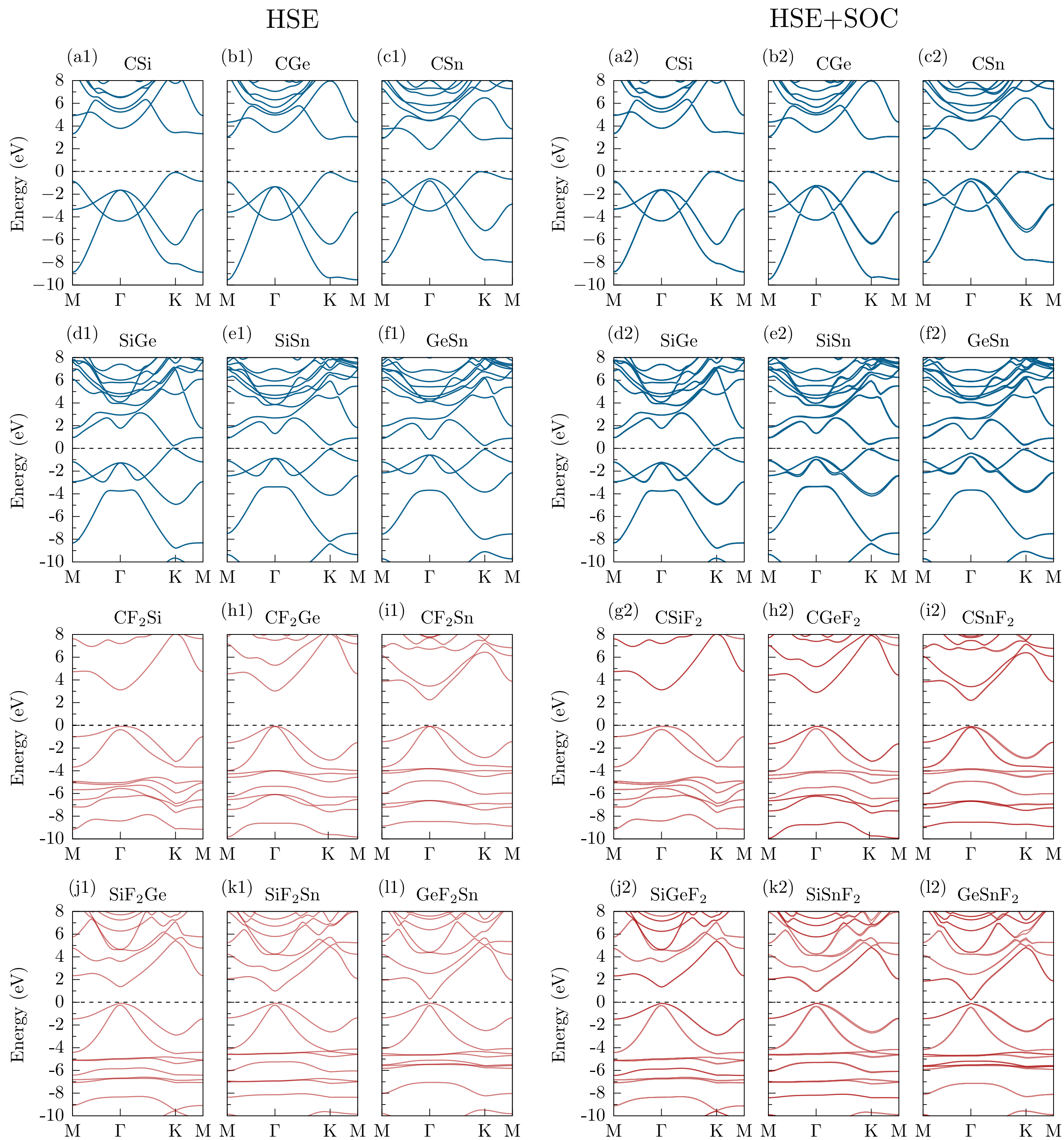

FIG. S1. HSE06 (a1-l1) and HSE06+SOC (a2-l2) electronic band structures of XY (blue) and XYF_2 (red) systems. All band structures were aligned with respect to the Fermi level.

PARTIAL FLUORINATION

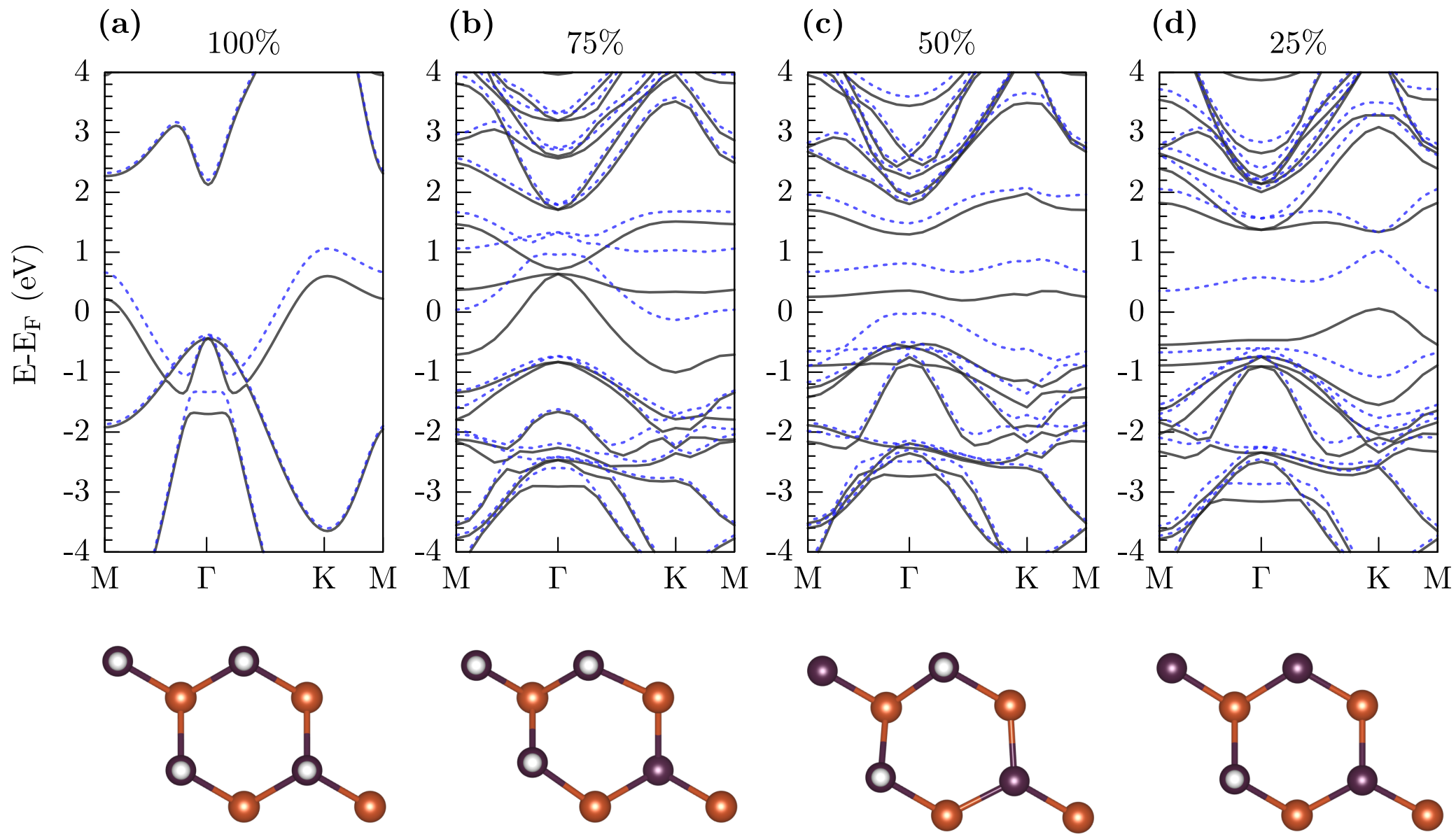

FIG. S2. Spin-resolved electronic band structures of SiGe monolayers fluorinated on a single side (Si-atoms only) with fluorine coverages of (a) 100%, (b) 75%, (c) 50%, and (d) 25%. The corresponding top-view atomic configurations are displayed below each band structure. Silicon, germanium, and fluorine atoms are represented by blue, gray, and white spheres, respectively. Spin-up and spin-down bands are denoted by black solid and blue dashed lines. For comparison, all band structures are referenced to the Fermi level.

We investigated the fluorination of the SiGe monolayer considering adsorption on only one side of the sheet and at different fluorine concentrations. The SiGe monolayer was selected as a representative case because it exhibits one of the strongest responses to fluorination among the studied compounds. In particular, the band gap increases significantly from 0.19 eV in the pristine monolayer to 1.43 eV in the fully fluorinated $SiGeF_2$ structure. This pronounced electronic modulation makes SiGe an ideal candidate for evaluating the effects of partial and one-sided fluorination.

First, the formation energy of a single fluorine adatom was calculated according to:

$$E_f = E_{\text{SiGe+F}} - E_{\text{SiGe}} - \frac{1}{2}E_{\text{F}_2}, \tag{S1}$$

where $E_{\text{SiGe+F}}$ is the total energy of the fluorinated system (with F adsorbed on either the Si

or Ge site), E_{SiGe} is the energy of the pristine monolayer, and E_{F_2} is the energy of an isolated fluorine molecule. The calculated formation energies indicate that fluorine adsorption is energetically more favorable on the Si site than on the Ge site, with $E_f^{\mathrm{Si}} = -3.49$ eV and $E_f^{\mathrm{Ge}} = -2.40$ eV. Therefore, subsequent calculations were performed considering fluorine adsorption on Si atoms.

To assess the effect of partial fluorination on the Si sites, three configurations with intermediate fluorine coverages (25%, 50%, and 75%) were considered alongside the fully functionalized monolayer (100%), as shown in Fig. S2. For this purpose, a $2 \times 2 \times 1$ supercell of the SiGe monolayer was constructed and fully relaxed, until the residual Hellmann–Feynman forces become smaller than 10^{-3} eV/Å, for each concentration. The electronic self-consistent calculations are converged to an energy tolerance of 10^{-8} eV

The electronic band structures presented in Fig. S2 were obtained using the HSE hybrid functional. The calculated band structures reveal that fluorination modifies the electronic properties of the SiGe monolayer depending on the adatom coverage. At lower coverages (25% and 50%), the system exhibits a narrow bandgap character, with indirect energy gaps of 0.07 eV and 0.04 eV, respectively. When the F coverage on the Si sites increases to 75%, a transition to a metallic state occurs, as evidenced by the energy bands crossing the Fermi level ($E - E_F = 0$ eV). This metallic behavior is maintained at 100% coverage. Furthermore, the splitting between the spin-up (solid curves) and spin-down (dashed curves) channels at both 75% and 100% coverages indicates that the spin-polarized character persists in these metallic phases. These findings demonstrate that functionalization of the Si sites enables tuning of the SiGe monolayer from a narrow-gap semiconductor to a spin-polarized metal as the coverage increases.
